# The interplay between the entrepreneurial leadership identity, entrepreneurial leadership competency and venture growth intentions of women in rural Australia

**DOI:** 10.1371/journal.pone.0296865

**Published:** 2024-02-02

**Authors:** Purushottam Dhakal, Retha Wiesner, Tek Maraseni

**Affiliations:** 1 School of Business, University of Southern Queensland, Toowoomba, Queensland, Australia; 2 Centre for Sustainable Agricultural Systems, University of Southern Queensland, Toowoomba, Queensland, Australia; Sichuan Agricultural University, CHINA

## Abstract

Cultivating business growth intentions in rural, regional, and remote women entrepreneurs is crucial, considering the unique challenges they face in rural areas. The growth intentions of rural, regional, and remote women entrepreneurs remain understudied. This study pioneers research on the interplay between entrepreneurial leadership competency, identity, and growth intentions of rural, regional, and remote Australian women. We surveyed rural, regional, and remote women entrepreneurs in Queensland, Australia, using structural equation modeling for analysis. Results revealed a positive relationship between entrepreneurial leader identity, business growth intentions, and entrepreneurial leadership competency. Moreover, entrepreneurial leadership competency positively correlated with growth intentions. The study indicated that entrepreneurial leadership competency partially mediates the link between identity and growth intentions. This research addresses a theoretical gap by introducing a new model showcasing the relationships between entrepreneurial leadership identity, entrepreneurial leadership competency, and venture growth intentions. From a practical standpoint, our findings strengthen the business case for improving tailor-made rural, regional, and remote entrepreneurial development programs.

## Introduction

Women entrepreneurs have a significant impact on the growth of small and medium-sized businesses in both developing and developed economies [1–3]. However, the number of women entrepreneurs in rural, regional and remote (RRR) areas globally is significantly lower compared to both male entrepreneurs in rural areas and women entrepreneurs in metropolitan areas. This is also the case in the Australian entrepreneurship ecosystem where women account for only 30% of all entrepreneurs and less than 13% of all women entrepreneurs [4, 5]. This highlights the necessity for a more comprehensive comprehension of the entrepreneurial practices of this particular context.

Furthermore, women’s entrepreneurship in rural, regional, and remote (RRR) regions in Australia, as well as in many other nations, has historically originated from the agricultural sector. In these places, women generate income for their households by engaging in entrepreneurial activities outside of farming. Nevertheless, there is a lack of clarity regarding the specific entrepreneurial activities of these women, the elements that can either facilitate or impede this activity in RRR communities [6] and the factors that impact their intentions to grow their businesses.

Understanding how women entrepreneurs perceive entrepreneurship and business growth is vital to overcoming gender-related challenges and advancing their careers. Women are less inclined to seek entrepreneurial careers compared to men due to their perception of lacking the requisite entrepreneurial skills, knowledge [7, 8], and familiarity with entrepreneurial responsibilities [9]. Wiesner [10] verified the existence of these self-imposed limitations that Australian RRR women entrepreneurs hold. It is therefore crucial to investigate the distinct characteristics of RRR women entrepreneurs in order to comprehend their perspectives on various elements of entrepreneurship and business growth. This understanding will help them overcome gender-related obstacles and progress in their entrepreneurial leadership careers.

The objective of this study is to examine the interplay between the entrepreneurial leader identity, entrepreneurial leadership competency and venture growth intentions in the context of women entrepreneurship in rural, regional and remote Australia. While previous studies have separately examined the topics of entrepreneurial leadership, entrepreneurial competencies, and venture growth in relation to women entrepreneurship, this study takes a unique approach by considering how these factors relate to each other.

Although there continues to be much debate about the definitions of the main constructs examined in this study, we drew on the following definitions to define the main measurement constructs in this study: Entrepreneurial leadership is defined as those individuals who recognize potential opportunities to exploit them by adding value and influence both internal and external people by building a broad base of support for their venture vision [11–13] We draw on Dhakal, Wiesner [5] work to define Entrepreneurial Leader Identity as a concept related to one’s perception of oneself as an entrepreneurial leader. Entrepreneurial growth intention is defined by Dutta and Thornhill [14] as “an entrepreneur’s goal or aspiration for the growth trajectory she or he would like the venture to follow”. Entrepreneurial leadership competence [15] is defined in this paper as the cognitive abilities that enable an individual to successfully manage and direct entrepreneurial ventures. It is the ability to effectively cope with the inherently uncertain and ever-changing contexts of entrepreneurship, make decisions under ambiguous and incomplete information conditions, inspire others by motivating and enthusing them, in addition to encouraging innovation and growth in an entrepreneurial firm.

The self-identity of RRR women entrepreneurs as entrepreneurial leaders and their competency to execute entrepreneurial activity, which drives their aim to build their ventures, remains unknown, despite their contribution to household revenue through off-farm entrepreneurial activity [1]. Moreover, despite the extensive research on the drivers of small business growth and the factors influencing business growth intentions, there remains a theoretical gap in understanding whether women entrepreneurs, specifically those from underrepresented contexts such as rural, regional and remote, perceive business growth as both desirable and achievable. Due to the limited understanding of RRR women’s growth ambitions, there is a dearth of conceptualization of explanatory growth theories related to their entrepreneurship [16, 17].

This study also addresses a practical gap. Due to a limited understanding of how RRR women entrepreneurs perceive themselves as entrepreneurial leaders and how this impacts the growth of their ventures, there is a distinct lack of programs that specifically address the intersection of entrepreneurial leadership, entrepreneurial leadership competency and enhancing the venture growth intentions of RRR women entrepreneurs. Enhanced comprehension of the interplay between these variables can guide government policy choices on funding initiatives aimed at bolstering the number of female entrepreneurial leaders. This can be achieved through the implementation of customized entrepreneurial leadership development and venture growth programs [18].

However, the question ‘why is entrepreneurial leadership identity relevant to the intention to grow the venture’ could be asked? Essers and Benschop [19] stress the importance of entrepreneurial leadership identity for detecting and exploiting opportunities in the market. Entrepreneurial identity crucially impacts the vision and strategic trajectory of entrepreneurs. Additionally, Down and Warren [20] illustrate the essence of developing an entrepreneurial identity within the individual and the business in order to gain awareness during the start-up process.

The role of entrepreneurial leadership identity in establishing the entrepreneur’s and venture’s credibility and legitimacy is emphasized [20]. They assert that an entrepreneurial leadership identity that is strong enough to be generalized across entrepreneurial roles can impact how the business is perceived in terms of legitimacy and development. Werthes, Mauer [21] findings suggest that the existence of a clear entrepreneurial leadership identity may contribute remarkably to one’s belief in personal capabilities and effectiveness of engagement in entrepreneurship.

Furthermore, by using the idea of ’discourse of enterprise’, Anderson and Warren [22] analyze the relationship between entrepreneurial identity and the broader environment. They found that identity as an entrepreneurial leader affects how a person interacts with the external environment and the broader entrepreneurial ecosystem, thus illustrating the importance of entrepreneurial leadership identity and its impact on influencing how stakeholders and the wider entrepreneurial environment are likely to interact. According to Sweida and Reichard [23], women involved in high-growth entrepreneurship have distinct attitudes and identities. They view impediments as opportunities to conquer and associate their firms with a positive self-image and identity.

For a number of reasons, it is essential for rural women entrepreneurs to acquire entrepreneurial leadership competencies. According to Cogliser and Brigham [24], entrepreneurs are successful in ventures because they possess certain key characteristics, namely technical knowledge and expertise and creative/innovative qualities. Entrepreneurial leadership competency, therefore, plays a pivotal role in influencing entrepreneurial performance. Developing entrepreneurial leadership competence can improve the technical and creative abilities required for entrepreneurial success as it assists in the achievement of personal and professional goals. RezaeiZadeh, Hogan [25] argue for the crucial interrelation between entrepreneurial skills, and the diverging impact of these skills among different groups and the geographical contexts. This highlights the importance of developing a broad range of skills to succeed in entrepreneurship [25]. The emphasis is on the importance of competence in entrepreneurial leadership in developing the broad range of skills and capabilities needed to effectively undertake the challenges of entrepreneurship. Moreover, Patterson, Mavin [26] suggest that, in order to be considered a credible and trustworthy leader in entrepreneurship, women must effectively manage stereotypically feminine and masculine prescriptive expectations. Often, women who fail to live up to prescriptive expectations are perceived to be less competent and trustworthy, irrespective of the expectations to which they supposedly fail to conform [26]. However, owing to the lack of knowledge of RRR women’s entrepreneurial leadership competencies, it is very difficult for RRR women entrepreneurs to effectively manage these stereotypical prescriptive expectations. This signifies a need for research, not only into their entrepreneurial identity but also their entrepreneurial leadership competencies in order to create new ways of thinking about entrepreneurial leadership identity and competency within the RRR women entrepreneurship context.

Examining the impact of rural Australian female founders’ entrepreneurial leadership (EL) identity and EL competency on their venture growth intentions is therefore important for several reasons. First, women entrepreneurship has become a critical component of economic development in many regions, and the growth of women-led businesses can have a significant impact on the economic growth of a region, especially in rural areas [27]. However, the phenomenon of business growth has been less explored in female-founded ventures [28], particularly in rural Australian female-founded ventures. It is important to understand the factors influencing the growth intentions of female founders in rural regions owing to the unique challenges and barriers these female founders face. Female founders in rural Australia areas face limited job opportunities compared to their urban and male counterparts, prompting them to seek entrepreneurial off-farm income and diversify on-farm activities, often amidst natural disasters like flooding and bushfires [29]. They also face cultural barriers due to male-dominated norms, limited access to capital, and insufficient local business education, leading to their under-representation in business and government [6, 30]. They face the challenge of distance to main centers and the lack of reliable internet; access to entrepreneurship development services; like-minded peers, networks, mentors; and confidence [5, 10]. Women in these areas, therefore, need to constantly act not only as entrepreneurs but also as entrepreneurial leaders by creating, identifying, and exploiting entrepreneurial opportunities and add value through enacting the challenges of communicating a vision and influencing others to help them realize it [11–13, 31, 32]. This study provides insights into how they can develop their EL identity and competencies to become stronger entrepreneurial leaders in their business and communities.

Second, because entrepreneurial leadership is a critical factor in the success of a venture [33], by studying the impact of entrepreneurial leadership identity and competency on venture growth intentions, we can better understand the factors that contribute to venture success.

Third, there is a need to better understand the role of entrepreneurial leadership identity and competency in fostering innovation and creativity in rural areas, which can offer unique opportunities for innovative and sustainable entrepreneurship, promoting the growth of women-led ventures and supporting economic development in rural and regional areas [13, 34].

Finally, understanding the role of entrepreneurial leadership identity and competency in promoting the growth of women-led ventures in rural areas can help to develop targeted interventions that can support gender diversity in entrepreneurship [35].

This study fills several research gaps. Firstly, there is limited research regarding the relationship between entrepreneurial leader identity and venture growth intentions of women entrepreneurs in rural, regional, and remote Australia. Additionally, less than six percent of all entrepreneurship research focuses on women entrepreneurs [36], making it a theoretical contribution to the domain of RRR women entrepreneurs. This study fills this gap by developing a new model that shows the relationships between EL identity, EL competency, and venture growth intentions.

Secondly, the growth intentions of rural female founders is understudied and challenging, given the combined factors of gender and rural location. Understanding the relationship between EL identity, competencies, and venture growth intentions is crucial, given the unique challenges and opportunities faced by these entrepreneurs.

Thirdly, this study fills a gap in the literature on entrepreneurial leadership (EL) by focusing specifically on RRR women’s EL, as this is the first study to examine the relationship between EL competency, EL identity, and growth intentions in this context.

Fourthly, our study fills a research gap on the measurement of EL by adapting the initial scale of Bagheri and Harrison [37] to enable RRR women entrepreneurs to reflect on their own EL competency, rather than relying on followers’ perspectives, which is the approached followed in other studies. This approach provides new insights into measuring EL in a RRR setting and for women entrepreneurs.

Finally, this study fills a gap in understanding the business growth phenomenon of women entrepreneurs to a greater extent [36]. This enhanced understanding helps build the business case for government and entrepreneurship development agencies to fund practical tailormade rural women EL development programs in which participants could enhance their competencies and develop their EL identities and venture growth intentions.

This study not only aims to deepen our understanding of women’s entrepreneurship but also seeks to provide practical implications for government and entrepreneurship development agencies. By investigating the interplay between entrepreneurial leader identity, competency, and venture growth intentions, this research aims to support the development of tailored programs that enhance competencies and foster entrepreneurial leadership among rural women, contributing to their success and the overall economic growth of the region.

## Hypothesis

Building on Harrison, Leitch [38] and Dhakal, Wiesner [5], we argue that contextualizing gender is essential to understanding entrepreneurial leadership (EL) in its entirety. Examining EL separately from its context is flawed as it fails to consider how the context shapes the opportunities and challenges experienced by women entrepreneurs due to spatial and institutional norms [39].

Some researchers argue that the desire to grow a business is inherent in entrepreneurs [40], but it has been shown that not all entrepreneurs intend to grow their business, especially women entrepreneurs [41, 42]. Women-owned businesses also tend to have lower survival rates, be less profitable, and employ fewer staff than those owned by men [43, 44], partly due to socialization influences on decision-making and work-family pressures [28, 45–47]. Additionally, contextual variables such as low self-efficacy and undervaluation of their work can influence women entrepreneurs to start businesses in low-growth industries and opt against scaling their ventures [48, 49]. Thus, the intentions to grow a business often differs between male and female founders.

Shepherd and Haynie [50] found that an entrepreneur’s identity is tied to decisions such as the growth of established ventures. EL Identity refers to one’s perception of oneself as an entrepreneurial leader, distinct from one’s identity as an entrepreneur [5]. Identity significantly influences behavior and is an integral part of the process of becoming a leader [51, 52].

In view of these assertions, the first research question we posed was whether the ‘EL identity’ of RRR women entrepreneurs impacts their intentions to grow their ventures. Our first hypothesis tested was, therefore:

*H1*: *The entrepreneurial leader identity of women entrepreneurs of RRR Australia positively affects venture growth intentions*.

This hypothesis is rooted in the idea that self-belief of RRR women entrepreneurs in their ability to perform specific tasks (Self-efficacy theory), observing and modelling the behavior of others (Social cognitive theory); and deriving a sense of meaning and purpose from their identity as a leader (Identity theory), can all have a significant impact on setting ambitious goals and pursuing venture growth with confidence and determination [53–55].

Moreover, the development of a leader’s identity, or sense of self, is unavoidable in leadership development [56]. In several studies, self-identity has been a predictor of intentions and actions, including those that account for prior behavior [e.g. 57–59]. Lord and Hall [60] assert that the identity of a leader aids in developing leadership competencies and the latter may help women entrepreneurs who possess strong entrepreneurial leadership competency to better utilize their skills and knowledge to drive venture growth. Leader identity also inspires a person to build leadership competency [61], and Miscenko, Guenter [62] found a positive relationship between leader identity and leadership competency.

Therefore, the second research question we posed was whether ‘EL identity’ impacts the ‘EL competency’ of RRR women entrepreneurs. Our second hypothesis was, therefore,

*H2*: *The EL identity of RRR women positively affects their EL competency*.

This hypothesis draws on the Human Capital Theory [63] which suggests that an individual’s skills, knowledge, and experience are critical determinants of their productivity and success.

Researchers have studied various competencies in the context of entrepreneurship, including entrepreneurial competencies, management competencies, social competencies, emotional competencies and leadership competencies [64–66]. These competencies have been linked to entrepreneurial growth and performance; specifically, the perception of competencies has been found to impact intentions [67, 68]. Other studies link entrepreneurial competencies and leadership competencies to entrepreneurial intentions [64, 69–71]. Moreover, researchers have found a positive relationship between competencies and growth aspirations [72–75]. Traditionally, researchers have studied entrepreneurial competencies and leadership competencies separately, however more recently, they have started defining “EL competency” as a single construct and argued that this competency is essential for entrepreneurial development [33, 76–78]. Therefore, because entrepreneurial competency and leadership competency have been found to link to entrepreneurial intentions, our third research question asked whether the EL competency of RRR women have the potential to impact entrepreneurial growth intentions. Our third hypothesis is, therefore:

*H3*: *The EL competency of RRR women entrepreneurs is positively related to their venture growth intentions*.

This hypothesis is based on not only the Self-efficacy theory [54], but also the Self-determination theory [79], to postulate that when female founders intrinsic values, interests and passion for their venture are served by growing their ventures, they are likely to be motivated and pursue venture growth.

Furthermore, an enhanced understanding of whether the EL competency of RRR women mediates the link between their EL identity and their venture growth intentions can help to generate awareness and empower these women to advance their EL identity, stimulate their will to grow their ventures and overcome the obstacles they face. Because there is still a knowledge gap regarding EL competency’s role in forming venture growth intentions, we asked whether the EL competency of RRR women mediates the relationship between their EL identity and venture growth intentions. Therefore, our final hypothesis is:

*H4*: *Australian RRR women entrepreneurs’ EL competency mediates the relationship between EL identity and venture growth intentions*.

## Materials and methods

### Data collection and sampling

Data for this research were collected via an online survey of 99 women entrepreneurs in rural, regional, and remote Queensland, Australia. This study is part of a larger funded project, The WiRE (women in rural, regional and remote enterprises) Program, which aims to assist women in RRR enterprises to start and grow their business. The population we wish to make inferences about are, thus Australian RRR women entrepreneurs. There are 452,000 small businesses in Queensland, Australia. Of these small businesses, 158,200 (35%) are owned or run by women. Our focus is to target the population of women entrepreneurs residing in remote or regional Queensland, which accounts for 33 percent (52,206) of the small women-owned businesses in the region. Due to the lack of an existing comprehensive database containing contact information for all women entrepreneurs in the remote or regional areas of Queensland, it was not feasible to access the entire population. Additionally, reliable information regarding the exact location and numbers of this population was not available.

To address this challenge, we adopted a research methodology in line with the work of Kille, Wiesner [4]. Specifically, we employed typical case, purposive sampling, which is a non-probability or non-representative sampling technique used when it is not possible to obtain a representative sample. Our focus was on capturing the typicality of study participants and contexts rather than achieving representativeness. While our findings cannot be generalized to the entire population, they serve as an illustration based on similar samples and enable comparisons with other relevant studies.

Initially, an email invitation to participate in the survey was sent to women entrepreneurs subscribed to the only Queensland RRR women entrepreneurship capacity building program, The WiRE Program. Then, an email was sent to several women networks and associations actively operating in RRR Australia, requesting them to invite members who are women entrepreneurs to participate in the survey. Potential respondents were invited to click on a survey link to fill out the survey.

Prior to filling out the survey, ethical consent was acquired from all participants through an online procedure, which involved participants clicking on the "agree" button. By clicking on the "agree" button, participants indicated their understanding of the provided information, expressed their voluntary willingness to participate, and affirmed that they were at least 18 years of age. This study received approval from the University of Southern Queensland Human Research Ethics Committee.

One-hundred-and-nine survey responses were received after multiple reminders. We excluded ten respondents owing to missing data, with the remaining 99 usable surveys included in the analysis. The survey was conducted from February 9^th^, 2022 to 31^st^, March 2022, and we assume the response rate would have been higher if RRR Australia had not been trying to cope with the reverberations of COVID and extreme flooding in RRR areas at the time of distributing the survey, which significantly negatively impacted small businesses in these areas, in particular [80].

The survey questionnaire was pretested with 10 participants in the study area to check the content validity and appropriateness of wording, formatting, and sequencing of questions. The questions were refined based on the outcomes of the pilot. Feedback on the questions and scales was also sought from a panel of four senior entrepreneurship and leadership academics and three entrepreneurship development experts. The measurement constructs measured in the survey included: Entrepreneurial leader identity (ELI), EL competency (ELC) and Venture Growth intentions (GI). The construct ELI was measured by four items adapted from [81]: “Developing and nurturing a venture/business is an important part of who I am (EL1)”, “I think of myself as an entrepreneur (EL2)”, “I think of myself as a leader (EL3)” and “When I describe myself, I would include the word leader (EL4)”.

To assess Entrepreneurial Leadership Capability (ELC), we utilized Bagheri and Harrison’s [37] EL Leadership Scale, which captures ELC from the perspective of followers. We adapted this survey to enable respondents to reflect on their EL behavior. The ELC is a higher-order reflective-reflective construct that measured seven lower-order components, including: Framing Challenges (FC) (5 items); Absorbing Uncertainty (AU) (4 Items); Underwriting (UW) (5 items); Building Commitment (BC) (5 items); Defining Gravity (DG) (4 items); Opportunity Identification and Exploitation (OIE) (11 items); and Orientation towards Learning (OTI) (5 items).

A single item, "My intention is to grow my venture as large as possible, " measured respondents’ growth intentions (GI). This scale was adapted from Edelman, Brush [82].

Measurement items were measured by using a 5-point Likert scale, where 1 is “strongly disagree” and 5 is “strongly agree”. The reliability and validity of measurement scales are discussed in the measurement model section.

### Data analysis method

To estimate the parameters of the mediation model, the partial least squares—structural equation modelling (PLS-SEM) method was implemented using Smart PLS 3 software. A PLS-SEM is a variance-based estimating method that examines the reliability and validity of constructs and evaluates the relationships between the constructs [83]. PLS-SEM’s advantage lies in its flexibility with less strict assumptions, allowing researchers to apply the method in diverse settings with smaller sample sizes. However, this flexibility comes at the cost of limited model fit indices, making the assessment of overall goodness of fit less straightforward compared to covariance-based SEM.

**Fig 1:**
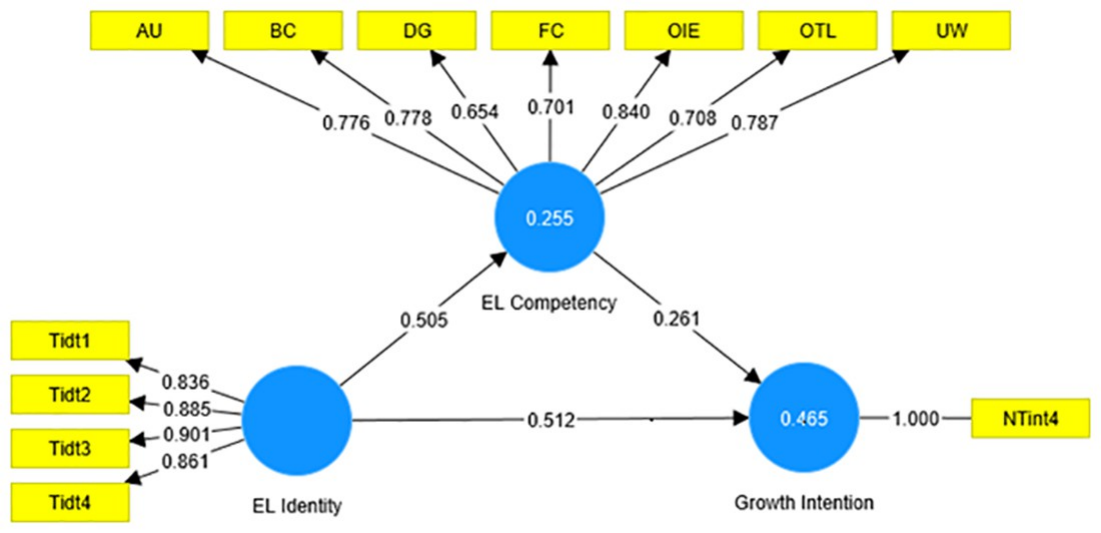
Structural model of the interplay between EL identity, EL competency, and growth intentions of women in rural Australia.

As our model consists of a higher-order construct ELC, we utilized the disjoint two-stage approach to evaluate our model. In the first stage, we created a model connecting the endogenous and exogenous constructs to the lower-order component of the Entrepreneurial Leadership competency construct. We first evaluated the quality of the measurement model for the lower-order components by assessing factor loadings, reliability and validity. After confirming the quality of the measurement model, we computed the latent variable score for the lower-order component of ELC. In the second stage, we created indicators for the higher order of ELC, using the latent variable scores obtained from the first stage, after which we created the second stage model. In this stage, we first assessed the quality of the higher-order measurement model, and then proceeded to hypothesis testing. The study tested the proposed hypotheses by using a structural model. To examine the proposed relationships, the study tested the mediation model by applying Preacher and Hayes’ approach by following the steps explained by F. Hair Jr, Sarstedt [84]. First, we examined the direct effect between ELI and GI by applying the bootstrapping procedure with 5000 sub-samples. After confirming the presence of the direct effects, in the second step, we included ELC as a mediator in the model (Fig 1). Then we examined the relationship between ELI, ELC and GI.

## Results

### Measurement model

#### Lower order measurement model

All the factor loadings for the lower-order constructs were greater than 0.5 (Table 1). This demonstrates that the indicators effectively capture the variance of their respective constructs. Cronbach’s alpha and composite reliability (CR) were higher than 0.7 (Table 1), which fulfilled the recommended threshold value [85]. These measures ensure internal consistency and reliability of the lower order measurement model. Convergent validity was acceptable because the Average Variance Extracted (AVE) was over 0.5 (Table 1). This indicates that more than half of the variance in each construct is explained by its indicators, confirming convergent validity of lower order measurement model.

**Table 1:** Loading, reliability, and validity statistics of lower-order components.

Constructs/ indicators	Loadings	*Composite reliability*	*Average Variance Extracted*
AU		0.8	0.577
AU1	0.857		
AU2	0.814		
AU3	0.579		
BC		0.838	0.571
BC2	0.774		
BC3	0.526		
BC4	0.886		
DG		0.835	0.717
BC5	0.789		
DG2	0.853		
DG4	0.840		
FC		0.832	0.555
FC2	0.630		
FC3	0.712		
FC4	0.812		
FC5	0.810		
OIE		0.879	0.514
OIE1	0.648		
OIE10	0.635		
OIE2	0.800		
OIE3	0.845		
OIE4	0.699		
OIE5	0.747		
OIE8	0.610		
OTL		0.823	0.541
OTL2	0.626		
OTL3	0.700		
OTL4	0.718		
OTL5	0.875		
UW		0.831	0.556
UW1	0.703		
UW2	0.866		
UW4	0.798		
UW5	0.586		
ELI		0.926	0.759
Tidt1	0.839		
Tidt2	0.879		
Tidt3	0.903		
Tidt4	0.862		
NTint4	1.000	1.000	1.000

Discriminant validity was confirmed by the Heterotriat-Monotrait ratio (HTMT) of correlation with values below 0.9 (Table 2) which satisfied the recommended threshold [86], providing evidence that the lower order constructs are distinct from each other. These values of internal consistency, convergent validity, and discriminant validity assertained the quality of lower order constructs.

**Table 2:** Discriminant validity assessment of lower order construct using the HTMT criterion.

Constructs	AU	BC	DG	FC	GI	ELI	OIE	OTL	UW
AU									
BC	0.742								
DG	0.778	0.773							
FC	0.709	0.715	0.379						
GI	0.505	0.323	0.38	0.466					
ELI	0.473	0.334	0.456	0.312	0.679				
OIE	0.897	0.688	0.803	0.65	0.611	0.589			
OTL	0.472	0.703	0.511	0.487	0.289	0.378	0.6		
UW	0.808	0.815	0.47	0.845	0.374	0.457	0.667	0.653	

#### Higher order measurement model

All the factor loadings for the higher order construct were greater than 0.5 (Table 3). This demonstrates that the indicators effectively capture the variance of their respective higher order constructs. The Cronbach’s alpha and composite reliability (CR) scores of the ELC construct were higher than 0.7 (Table 3), which fulfil the recommended threshold value [85]. These measures ensure internal consistency and reliability of the higher order measurement model. The convergent validity of the higher-order construct was acceptable because the Average Variance Extracted (AVE) was over 0.500 (Table 3). This indicates that more than half of the variance in each construct is explained by its indicators, confirming convergent validity of the higher order measurement model.

**Table 3:** Loading, reliability, and validity statistics of higher-order components of EL competency.

	*Loadings*	*Cronbach’s alpha*	*Composite reliability*	*Average Variance Extracted*
ELC		0.871	0.900	0.565
AU	0.776			
BC	0.778			
DG	0.654			
FC	0.701			
OIE	0.840			
OTL	0.708			
UW	0.787			

The Heterotriat-Monotrait ratio (HTMT) of correlations with values below 0.9 (Table 4) ascertained discriminant validity for the higher-order construct ELC, providing evidence that the higher order constructs are distinct from each other. These values of the internal consistency, convergent validity and discriminant validity ascertained that the quality of the higher order construct was sufficient.

**Table 4:** Discriminant validity assessment of higher order construct using the HTMT criterion.

	ELC	GI	ELI
ELC			
GI	0.538		
ELI	0.551	0.679	

#### Structural model

The results revealed that the direct effect between the constructs was positive and significant (β = 0.643, P<0.001; Table 5). The results support our first hypothesis that the EL identity of RRR women entrepreneurs has a positive effect on their venture growth intentions.

**Table 5:** Direct and indirect effect of ELI on GI.

	ß	95%CI	*T*	*P Values*
**Direct effect**				
ELI -> GI	0.643	0.485–0.759	7.637	0.000
**Indirect effect**				
ELI -> ELC -> GI	0.132	0.056–0.232	2.475	0.013

The results also support our second hypothesis that the EL identity of RRR women has a significant positive effect on their EL competency (β = 0.505, P<0.001; Table 6). Similarly, our third hypothesis was confirmed that the EL competency of RRR women entrepreneurs is significantly positively related to their venture growth intentions (β = 0.261, P = 0.008; Table 6). Moreover, the results revealed that the effect of the indirect path ELI to ELC to GI was positive and significant (β = 0.132, P = 0.013; Table 5). The results support our fourth hypothesis that the ELC mediates the relationship between ELI and GI. However, the mediating effect of ELC did not fully suppress the direct relationship between EL and GI, as the path from ELI to GI was significant and positive (β = 0.512, P<0.001; Table 6) on the presence of the mediating variable ELC. Hence, the mediating effect of ELC is partial.

**Table 6:** Path coefficients of the structural model of the interplay between EL identity, EL competency, and growth intentions of women in rural Australia.

Path	ß	95%CI	*T*	*p-value*
ELC -> GI	0.261	0.109–0.434	2.64	0.008
ELI -> ELC	0.505	0.422–0.613	8.74	0.000
ELI -> GI	0.512	0.485–0.759	4.546	0.000

#### Robustness checks

In our study, we implemented robustness checks to ensure the reliability and stability of our findings. To assess the precision of our parameter estimates, we employed bootstrapping as a resampling technique, generating 5000 bootstrap samples. The 95% confidence intervals (CI) obtained from these samples, as presented in Tables 5 and 6, offer a range within which the true population parameters are likely to lie, reinforcing the robustness of our results.

To gauge the explanatory power of the structural model depicted in Fig 1, we calculated the *R2* values, indicating the explained variance of the dependent constructs [87]. The *R2* values for both ELC (0.255) and GI (0.465) were higher than the minimum threshold of 0.10 [88], signifying the model’s ability to account for a significant portion of the variability in the dependent variable.

Last but not least, *Stone-Q2 Geisser’s* was used to assess the predictive value of the model [87]. All *Q2* values in the results were found to be greater than zero (see Table 7), indicating that the models have predictive value.

**Table 7:** Explanatory power and predictive value of the model.

*Constructs*	*R2*	*Q2*
ELC	0.255	0.133
GI	0.465	0.409

Last but not least, *Stone-Q2 Geisser’s* was used to assess the predictive value of the model [87]. All *Q2* values in the results were found to be greater than zero (see Table 7), indicating that the model’s capability to predict endogenous latent variables beyond the sample used for model estimation.

## Discussion

This study examined the interplay between EL competency, EL identity and growth intentions of RRR Australian women using a Partial least square structural equation model. Our findings supported our first hypothesis that RRR women entrepreneurs with a strong EL identity have a higher intention to grow their businesses. The present finding supports the conclusion reached by Rise, Sheeran [89] in a meta-analysis, suggesting that self-identity accounts for a significant portion of the additional variance in intentions. This result aligns with previous studies that have also identified self-identity as a predictor of behavioral intentions, as demonstrated in studies by Paquin and Keating [57], Carfora, Caso [58], and Obschonka, Silbereisen [59]. The results also support the growing evidence that the Theory of Planned Behavior (TPB) model ought to incorporate identity to more accurately predict individuals’ intentions [e.g. 90–92]. The TPB predicts an individual’s intentions to engage in a behavior at a specific time and place [93].

Our results also demonstrate that RRR women’s entrepreneurial leadership (EL) identity is significantly positively related to EL competency, supporting our second hypothesis that the EL identity of RRR women positively affects their EL competency. This finding corresponds with the results of [62], who found that the identity of a leader correlates with their leadership competency. Furthermore, our study establishes a positive association between EL competency and growth intentions, supporting our third hypothesis that the EL competency of RRR women entrepreneurs is linked to their venture growth intentions. These findings are in line with prior research conducted by Fuller, Liu [67] and Chang, Shu [68]. Our finding is important for developing EL competency of Australian RRR women as Lord and Hall [60] reported that self-identity not only influences the development of leadership competency, but also inspires a person to build leadership competency [61]. As mentioned earlier, addressing the development of the EL identity of Australian RRR women in current and future RRR entrepreneurship capacity-building programs is essential to enhance their EL competency.

Our findings support the notion that EL identity plays a role in learning EL competency as identity plays a significant role in influencing, molding, and choosing behavior [51, 52]. Competencies gained through learning experiences are reflected in their actions as entrepreneurs adapt their behavior in response to new information. Our findings indicate that EL identity, EL competency and growth intentions are related and that EL competency partially mediates the relationship between EL identity and venture growth intentions, in line with our fourth hypothesis that Australian RRR women entrepreneurs’ EL competency mediates the relationship between EL identity and venture growth intentions. Our mediation analysis found EL identity as a significant contributor to women entrepreneurs’ growth intentions owing to its direct and indirect relationship with the construct. One could therefore argue that in future research, EL identity ought to be included in models that examine the venture growth phenomenon. The relationships between all the measurement constructs in this study have not been explored previously; therefore, our study contributes to the EL literature by addressing a theoretical gap.

From a practical perspective, our study strengthens the case made by Birdthistle, Eversole [49], that clear attention needs to be paid to gendered dynamics in rural entrepreneurial ecosystems and for these ecosystems to be inclusive of women’s gendered experiences [94]. The constructs in this study were examined from participants’ own perspectives, allowing us to gain a close-up look at the self-perceptions of women entrepreneurs on the margins, concerning EL identity, EL competency and venture growth intentions. By doing so, we hope our findings contribute to creating a more inclusive entrepreneurial ecosystem for RRR women entrepreneurs in Australia.

## Practical implications

Our findings underpin the following practical implications:

First, designing and implementing entrepreneurial leadership (EL) advancement programs specifically tailored for RRR women entrepreneurs (RRR), can help foster their unique entrepreneurial leadership identities and competencies. A number of studies indicate the effectiveness of individualized support programs for women entrepreneurs in remote rural areas [1]. These programs should ideally be developed to enhance the entrepreneurs’ sense of being an entrepreneurial leader. This emphasis is important since our findings showed that a sense of entrepreneurial leadership identity has a direct positive effect on an aspiring entrepreneur’s intentions to grow their business.
Second, establishing mentoring relationships and exposing them to lessons learnt by successful female role models who exemplify their rural entrepreneurship may act as a crucial source of inspiration and backing. The support gained from this exposure provides an avenue for these entrepreneurs to cultivate their business identity while nurturing more ambitious goals for scaling up [95].
Third, creating bespoke networking and community forums dedicated solely towards RRR women in entrepreneurship could also strengthen their entrepreneurial leadership identity and intentions to grow their ventures. Such networks or communities could enable connections with like-minded individuals or industry professionals which might lead to potential collaborations that promote both business objectives along with driving policy changes—further strengthening the entrepreneur’s resolve while spurring growth strategies within their businesses [94].
Forth, targeted support from policy makers and organizations is required to promote the entrepreneurial identity and further the growth of women-run businesses in RRR Australia. Policy makers should play a proactive part by supporting rural-based female-owned enterprises through mitigating barriers faced by them whilst formulating measures addressing specific issues related primarily due to geographical constraints encountered by such ventures operating out of remote or regional areas [1, 96]. Therefore, tailoring policies based on distinctive needs they have in order overcome challenges effectively would be beneficial overall [4]. For example, initiatives designed around fostering better trading conditions generally coupled with incentives promoting RRR women-owned start-ups together with social upliftment programs aimed at gender parity in entrepreneurship, could be particularly impactful when dealing directly within RRR contexts.
Fifth, a deeper dive into understanding those parameters influencing success rates achieved especially amongst marginalized groups such as RR First Nation, Migrant and Refugee Women who show eagerness expanding their businesses whilst simultaneously overcoming challenges arising due to intersecting factors like race, ethnicity and gender bias within society [97]. This can provide important insights enabling the creation of effective policy interventions plus support mechanisms aimed towards achieving exactly those objectives as outlined above.
Sixth, it is important for the authorities and financial bodies to look into the financial barriers which are being faced by RRR women entrepreneurs and must allocate funds and resources to make it easier for the rural women entrepreneurs to access finance, for example offering loans on easy terms and conditions. This is supported by recent research, which shows that making credit more available is critical for overcoming the specific financial difficulties of rural women entrepreneurs [98]. By mitigating the financial barriers of RRR women entrepreneurs, an environment can be created in which they could more readily tap into potential growth opportunities available in their regions.

## Limitation

There are certain research methodological limitations that impede the applicability of our study’s results. The sample size of 99 respondents reflected the specific objectives of our investigation, however the small sample size pose constraints in relation to the external validity and generalizability of our findings beyond the studied population. The primary limitations arise because control variables were intentionally left out from the study’s analysis. This was motivated by the study’s exploratory nature and the small sample size, raising concerns about prioritizing simplicity as well as concerns over potential model overfitting tied to its small cohort size.

Leaving out control variables may limit the ability of the model to account for specific observed factors. Furthermore, owing to the unobserved variables, alongside potential measurement limitations and the context-specific nature of findings, care must be taken when generalizing our findings. Moreover, having utilized a cross-sectional framework in conducting the survey poses another limitation since it hampers determining cause-effect relationships among variables over time.

We recommend that future exploratory research endeavors incorporate larger and more diverse samples, control variables, and alternative research designs. This would allow for a broader understanding of the relationships under investigation while also addressing the acknowledged research limitations.

## Recommendations

In view of the cross-sectional design limitation of our study, future research should include longitudinal and experimental studies to explore the relationships between EL identity, EL competency, and growth intentions more comprehensively.

Furthermore, our study revealed that the EL competency of RRR women entrepreneurs only partially mediate the relationship between EL identity and entrepreneurial growth intentions. These findings indicate that other paths could be from EL identity to entrepreneurial growth intentions. We recommend that future research include other constructs to explore possible paths.

Our study is based on a specific demographic from RRR settings in Australia, which may differ from other situations. Consequently, future research should examine the measurement constructs in other geographic contexts.

Two additional areas that require further exploration is the gendered nature of entrepreneurial identity and its impact on women’s entrepreneurial experiences and growth intentions; and an exploration of how women incorporate their leadership identity with their ethnic background, as well as with their gender. The existence of these research gaps underscores the critical importance of considering the ways in which gender, ethnicity, and other social identities intersect and shape entrepreneurial leadership identity.

Despite these limitations, the findings of this study deepen our understanding of a new area of research in the RRR context and that there is much more to explore about the role that EL identity and EL competency play in the venture growth intentions of RRR women entrepreneurs.

## Conclusion

This investigation explored the complex interconnectedness of entrepreneurial leadership (EL) identity, EL competency, and venture growth intentions among RRR Australian women entrepreneurs utilizing a Partial Least Squares Structural Equation Model. The results lend strong credence to the central role that RRR women entrepreneurial leadership identity play in positively influencing their growth intentions, thereby augmenting existing evidence-based literature on self-identity and intentions.

On a practical note, our research highlights how vital it is to establish specific schemes and programs such as leadership training programs and mentorship opportunities along with networking and community platforms in order to boost both the confidence levels and skills among rural women entrepreneurs. These efforts are essential elements towards stimulating their ambitions to grow and expand their businesses. Policymakers also have an integral part in shaping a conducive entrepreneurship environment through gender-friendly policies designed to encourage business growth amongst rural women entrepreneurs. However, there were certain limitations inherent in employing a smaller sample size devoid of control variables as well as using cross-sectional design methods during data collection stage.

Looking ahead we propose more extensive longitudinal studies coupled with comprehensive experiments to determine cause-effect relationships while simultaneously investigating alternative routes linking RRR women entrepreneurs’ EL identities, EL competencies and their venture growth intentions. Furthermore, expanding research parameters across varying geographical settings and different socio-economic backgrounds will serve to enhance the generalizability of the findings. Despite the acknowledged limitations, the current study pioneers a novel exploration into RRR women entrepreneurship, offering fresh insights regarding the intricate interplay between various facets of RRR women entrepreneurship in rural, regional and remote Australia.

We appreciate the potential of our study to inform evidence-based practices and policies for RRR women entrepreneurs, contributing to a more inclusive entrepreneurial ecosystem in Australia.

Our collective aim is promoting deeper understanding nuanced dynamics associated gender-based differences empowering novice veterans alike navigate unfamiliar terrains successfully achieve desired outcomes. Addressing these limitations in future research will ensure a more comprehensive understanding of gender dynamics in entrepreneurship. Finally, this is a timely study in view of the lack of female EL role models in rural areas and women entrepreneurs questioning their confidence and abilities to grow their businesses. Our study answered the call to not only contribute to research on the gender gap in entrepreneurship but to be grounded in a gendered perspective that is constructed on a ‘female norm’ rather than how women entrepreneurship diverges from the male norm benchmark. Our research demonstrates that theoretical attention ought to be paid to tease out further the link between the EL identity, EL competency and venture growth intentions of RRR women entrepreneurs. Nevertheless, the results have shown that there is a clear case for policy development that advances RRR women entrepreneurs through strategies and pathways that develop the EL identity and EL competency of RRR women entrepreneurs to strengthen their confidence, skills and appetite to grow their ventures.

## Supporting information

S1 Data (XLSX)
